# The Canadian Heart Failure (CAN-HF) Registry: A Canadian Multicentre, Retrospective Study of Outpatients with Heart Failure

**DOI:** 10.1016/j.cjco.2024.09.014

**Published:** 2024-10-09

**Authors:** Dimitar Saveski, Melanie Kok, Stephanie Poon, Carlos Rojas-Fernandez, Sean A. Virani, George Honos, Robert McKelvie

**Affiliations:** a St Joseph’s Health Care, Western University, London, Ontario, Canada; b Novartis Pharmaceuticals Canada Inc., Montreal, Quebec, Canada; c Sunnybrook Health Sciences Centre, University of Toronto, Toronto, Ontario, Canada; d University of British Columbia, Vancouver, British Columbia, Canada; e Center Hospitalier de l'Université de Montréal, Montreal, Quebec, Canada

## Abstract

**Background:**

Guideline-directed medical therapy (GDMT) reduces events in patients with heart failure (HF) with reduced ejection fraction (HFrEF). Despite this impact, underutilization of GDMT persists. This report sought to describe HF management in Canadian outpatients treated at specialized HF clinics (HFCs).

**Methods:**

The Canadian Heart Failure (CAN-HF) study was retrospective and observational, and it included 1775 patients from 6 Canadian outpatient HFCs, from the period January 2017-April 2020.

**Results:**

We observed improvement in prescription rates in patients with HFrEF, between their first visit and their most-recent clinic visit, across all GDMT classes, in those who were followed at the HFC for ≥ 6 months. The largest prescription rate increases were observed for angiotensin receptor–neprilysin inhibitors and mineralocorticoid-receptor antagonists. However, more than half of the patients remained on angiotensin-converting enzyme inhibitors and/or angiotensin-receptor blockers, despite being symptomatic, according to their New York Heart Association class. Most patients (50%) were on triple therapy, as of their most-recent visit, with fewer (36%) on dual therapy, monotherapy (13%), or no GDMT (2%). Our data also suggest that patients who had been managed at the HFC for > 6 months had higher prescription rates of GDMT and were on higher doses of GDMT, compared to those who were new to the clinic. For patients with HF with preserved ejection fraction, few patients were on candesartan and less than half were on a mineralocorticoid-receptor antagonist.

**Conclusions:**

Our data from HFCs that in most cases were affiliated with academic centres compare favourably with data from other analyses of ambulatory patients with HFrEF, evidence that supports the use of a specialized patient-care model.

## Résumé

**Contexte:**

Le traitement médical fondé sur les lignes directrices (TMFLD) réduit les événements chez les patients atteints d’insuffisance cardiaque (IC) avec fraction d’éjection réduite (ICFER). Malgré cela, le TMFLD demeure toujours sous-utilisé. Ce rapport visait à décrire la prise en charge de l’IC chez des patients externes de cliniques spécialisées en IC.

**Méthodologie:**

L’étude CAN-HF (*Canadian Heart Failure*), une étude canadienne rétrospective et observationnelle sur l’insuffisance cardiaque, comprenait 1 775 patients traités dans 6 cliniques externes canadiennes spécialisées en IC entre janvier 2017 et avril 2020.

**Résultats:**

Nous avons observé une amélioration du taux de prescription dans toutes les classes de TMFLD entre la première visite et la plus récente visite chez les patients atteints d’ICFER traités durant au moins 6 mois à leur clinique d’IC. Les plus importantes hausses du taux de prescription ont été observées pour les inhibiteurs du récepteur de l’angiotensine-néprilysine et les antagonistes des récepteurs minéralocorticoïdes. Cependant, plus de la moitié des patients sont demeurés sous inhibiteurs de l’enzyme de conversion de l’angiotensine et/ou antagonistes des récepteurs de l’angiotensine, malgré la présence de symptômes, en fonction de leur classe NYHA (New York Heart Association). À leur plus récente visite, la plupart des patients recevaient une trithérapie (50 %), un moins grand nombre recevaient une bithérapie (36 %), une monothérapie (13 %) ou aucun TMFLD (2 %). Nos données indiquent également que les taux de prescription de TMFLD et les doses de TMFLD étaient plus élevés chez les patients pris en charge dans une clinique spécialisée en IC depuis plus de 6 mois que chez ceux qui étaient nouveaux dans la clinique. Dans le cas des patients atteints d’IC avec fraction d’éjection préservée, quelques-uns prenaient du candésartan et moins de la moitié prenaient un antagoniste des récepteurs minéralocorticoïdes.

**Conclusions:**

Nos données provenant des cliniques spécialisées en IC, qui étaient dans la plupart des cas affiliées à des centres universitaires, se comparent avantageusement avec des données d’autres analyses portant sur des patients ambulatoires atteints d’ICFER, ce qui milite en faveur de l’utilisation d’un modèle de soins spécialisés.

The burden of heart failure (HF) in Canada is significant. Approximately 750,000 Canadians live with HF, with 100,000 being newly diagnosed annually.^1^ HF is among the top reasons for hospitalizations nationally,^2^ with over 70,000 yearly admissions, and a 7-day median length of stay.^3^ Mortality and morbidity reductions in patients with HF with reduced ejection fraction (HFrEF) are well documented for angiotensin-converting enzyme inhibitors (ACEis), angiotensin-receptor blockers (ARBs), angiotensin receptor–neprilysin inhibitors (ARNis), beta-adrenergic antagonists (βbs), mineralocorticoid-receptor antagonists (MRAs), and sodium glucose cotransporter-2 inhibitors (SGLT2is).^4^^,^^5^ Multiple international evidence-based guidelines provide clear recommendations regarding the importance of optimizing medical therapy.^5^, ^6^, ^7^ Nevertheless, a recent Canadian study demonstrated that the 30-day HF readmission rate has remained at approximately 20% over the past decade.^3^ In part, this rate may be attributable to suboptimal use of guideline-directed medical therapy (GDMT).^8^^,^^9^

The Canadian Heart Failure (CAN-HF) Registry was designed to provide a contemporary description of HF management across the continuum of care, from inpatient to outpatient settings. Findings from the CAN-HF Registry have documented room for improvement in GDMT use at discharge following a hospital admission for acute HF (AHF).^10^^,^^11^ The objective of this CAN-HF Registry report is to describe HF management in patients treated at outpatient HF clinics (HFCs).

## Methods

### Design

The CAN-HF Registry design has been described previously.^10^ Briefly, the CAN-HF study was retrospective and observational, and it included consecutive patients with HF, comprising 2 cohorts, as follows: (i) inpatients admitted with AHF; and (ii) outpatients treated in HFCs.

The current analysis was limited to outpatients. The cohort consisted of patients with HF, aged 19-95 years, from 6 HFCs in urban sites in Quebec, Ontario, Manitoba, and British Columbia, Canada. The majority of HFCs (5 of 6) were affiliated with academic hospitals; 1 site was not. All sites were multidisciplinary clinics where patients were treated by HF specialists, although not all treating physicians were cardiologists. No exclusion criteria were applied. Data were collected between January 2017 and April 2020 and were abstracted from the medical record into a secure, Web-based, electronic data-capture platform designed by IQVIA (Kirkland, Quebec, Canada). Patient data reported by healthcare providers included patient demographics, medical history, HF medications, laboratory values, and left-ventricular ejection fraction (LVEF) from their most-recent HFC visit. The information in the medical record was used to classify patients as having HFrEF vs HFpEF. All information was captured from the patient’s medical record. Study-site research personnel received training from IQVIA on use of the electronic data-capture platform prior to the start of data collection. Participating sites obtained ethics review board approval prior to study commencement.

### Sample size and statistical analysis

Given the descriptive study design, no sample-size justification was performed. Each site was expected to identify 300 outpatients, for an intended sample size of 1800 patients. Descriptive statistics were used to report baseline characteristics and medical-therapy prescription. Categorical values were reported as frequencies with percentages, and continuous variables were reported as means with standard deviations or medians, with interquartile ranges (IQRs), based on the normality of the distribution. Basic adherence to GDMT was calculated for all patients, based on medication prescription as captured in the medical record.

Patients with HFrEF were classified further, based on the length of time they had been followed at the HFC, as follows: those who were followed for a minimum of ≥ 6 months (ie, allowing adequate time for optimization of medical therapy), vs those who were new to the HFC, defined as their having had an initial visit to the HFC or having been followed at the HFC for < 6 months. Ineligibility for medical therapy was tabulated, based on available testing and laboratory values (eg, LVEF, potassium, and estimated glomerular filtration rate), as previously reported for CAN-HF inpatients.^11^ Indication-corrected adherence to GDMT was calculated, as reported elsewhere,^12^ and it was calculated for the most-recent visit to the HFC, based on the laboratory values listed above. Patients were defined as being on monotherapy if they were prescribed medication from only a single GDMT class, dual therapy if they were prescribed 2 GDMT classes, and triple therapy if they were on all 3 GDMT classes.

Target doses for patients with HFrEF were defined based on Table 1 of the 2017 *Canadian Cardiovascular Society* (CCS) *Guidelines for the Management of Heart Failure*.^13^ A modified HF “collaboratory score,” similar to that proposed by Fiuzat and colleagues (2022),^14^ was used for patients who had complete dosing information available, and it was modified to align with the CCS guideline recommendations. Dosages that were < 50% of the target dose could not be assigned points in our study, as this information was not collected for all medication classes.

**Table 1:** Baseline characteristics

Characteristic	HFrEF	HFpEF
Total	**1319 (74.3)**	**447 (25.2)**
Age, y		
Median (IQR)	71.0 (17.0)	77.0 (18.5)
≤ 64	431 (32.7)	98 (21.9)
65–74	403 (30.6)	96 (21.5)
≥ 75	485 (36.8)	253 (56.6)
Sex		
Male	950 (72.0)	224 (50.1)
Female	369 (28.0)	223 (49.9)
Comorbidities		
Hypertension	758 (57.5)	293 (65.6)
Coronary disease	667 (50.6)	137 (30.7)
Atrial fibrillation	494 (37.5)	251 (56.2)
Diabetes	506 (38.4)	203 (45.4)
Chronic kidney disease	400 (30.3)	169 (37.8)
COPD	206 (15.6)	113 (25.3)
Anemia	216 (16.4)	97 (21.7)
Sleep apnea	170 (12.9)	98 (21.9)
Patient admitted for the following, in past 12 mo		
None of the below	685 (51.9)	231 (51.7)
Acute HF	371 (28.1)	144 (32.2)
ICD and/or CRT	47 (3.6)	2 (0.5)
Other conditions	305 (23.1)	109 (24.4)
Year of visit		
2017	23 (1.7)	6 (1.3)
2018	82 (6.2)	22 (4.9)
2019	1086 (82.3)	399 (89.3)
2020	128 (9.7)	20 (4.5)

Values are n (%), unless otherwise indicated.

COPD, chronic obstructive pulmonary disease; CRT, cardiac resynchronization therapy; HF, heart failure; HFpEF, HF with preserved ejection fraction; HFrEF, HF with reduced ejection fraction; ICD, implantable cardioverter defibrillator; IQR, interquartile range.

## Results

### Patient characteristics

Data from 1775 patients seen at HFCs were analyzed. Most patients (55.4%) were referred by a cardiologist; 81.4% of visits (n = 1445) were routine follow-up appointments. A classification of HFrEF vs HFpEF was documented in 74.3% and 25.2% of patients, respectively; LVEF was unknown in 9 patients. Baseline characteristics are reported in Table 1. The median age for patients with HFrEF was 71 years (IQR, 17); 67.4% were aged > 65 years; and 72.0% were male. Most patients (51.9 %) had had no hospital admission in the previous 12 months. The most-common reasons for admission in the prior 12 months were AHF (28.1%) and other conditions (23.1%). The most-common comorbidities were hypertension (57.5%), coronary artery disease (CAD; 50.6%), atrial fibrillation (37.5%), diabetes (38.4%), and chronic kidney disease (30.3%). Most patients had had their most-recent visit to the HFC in 2019 (82.3%). Patients with HFpEF tended to be older, were more likely to be female, and had higher rates of comorbidities, but lower rates of CAD, relative to those of HFrEF patients.

New York Heart Association (NYHA) class was documented in > 90% of patients. Most patients were in NYHA class II (> 50%) or III (> 20%; Table 2). The median LVEF, for patients with HFrEF vs HFpEF, was 33% (IQR, 17.0) and 60% (IQR, 8.5), respectively. Laboratory tests from the most-recent clinic visit were available for 97% of patients (Table 2).

**Table 2:** Most-recent evaluations and laboratory values

Evaluation	Patients with HFrEF	Patients with HFpEF
NYHA class	**1319 (74.3)**	**447 (25.2)**
I	180 (13.7)	44 (9.8)
II	707 (53.6)	240 (53.7)
III	301 (22.8)	116 (26.0)
IV	9 (0.7)	7 (1.6)
Missing or not measured	122 (9.2)	40 (9.0)
LVEF	**1318 (99.9)**	**445 (99.6)**
	33.0 (17.0)	60.0 (8.5)
Patients with an available laboratory test	**1279 (97.0)**	**433 (96.9)**
Serum sodium, mmol/L	**1265 (95.9)**	**426 (95.3)**
	140.0 (4.0)	140.0 (4.0)
Serum potassium, mmol/L	**1263 (95.8)**	**426 (95.3)**
	4.4 (0.6)	4.3 (0.6)
Serum creatinine, μmol/L	**1264 (95.8)**	**427 (95.5)**
	106.0 (58.3)	114.0 (67.5)
eGFR, mL/min per 1.73 m^2^	**912 (69.1)**	**290 (64.9)**
	57.9 (32.2)	49.0 (33.2)
Hemoglobin, g/L	**889 (67.4)**	**337 (75.4)**
	129.6 (20.0)	125.4 (19.5)
BNP, pg/mL	**113 (8.6)**	**45 (10.1)**
	413.0 (612.0)	311.0 (357.0)
NT-pro BNP, pg/mL	**108 (8.2)**	**46 (10.3)**
	1425.0 (2676.8)	1035.0 (2270.3)

For hemoglobin, lower row values are mean (standard deviation). For other categories with 2 rows, values in lower row are median (interquartile range). Other values are n (%).

eGFR, estimated glomerular filtration rate; HFpEF, heart failure with preserved ejection fraction; HFrEF, heart failure with reduced ejection fraction; LVEF, left-ventricular ejection fraction; NT-pro BNP, N-terminal pro-brain natriuretic peptide; NYHA, New York Heart Association.

### Prescription of guideline-directed medical therapies (GDMT) for patients with HFrEF

#### Patients followed at the HFC for ≥ 6 months

More than half of the patients diagnosed with HFrEF had been followed at the HFC for ≥ 6 months (n = 716; 55.1%). For this subset of patients, data from their most-recent visit to the HFC, as well as from their initial visit to the HFC, were available and were analyzed (see also Supplemental Fig. S1). The length of time between patients’ first visit and most-recent visit to the HFC varied, as follows: 6-12 months (218; 30.4%); 12-24 months (209; 29.2%); 24-36 months (111; 15.5%); and > 36 months (178; 24.9%).

**Figure 1:**
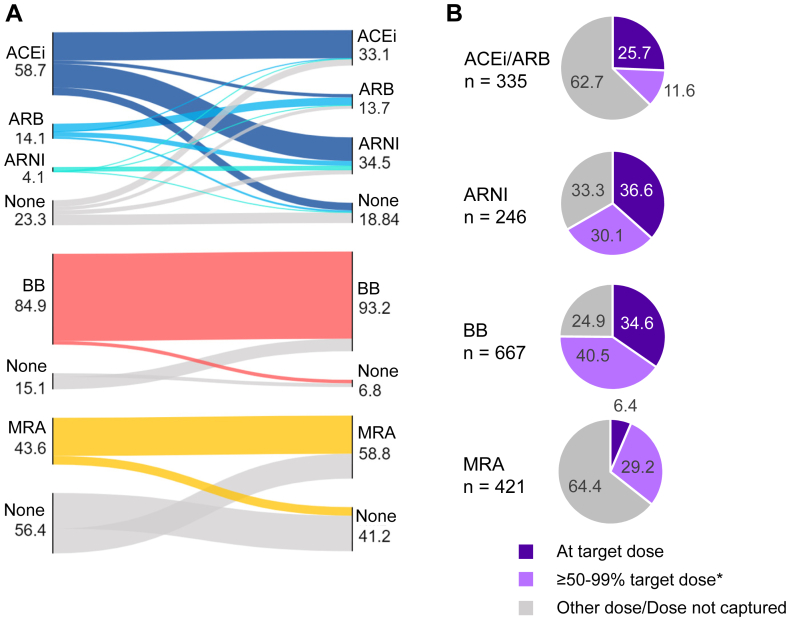
Guideline-directed medical therapy for patients followed at a heart failure clinic (HFC) for ≥ 6 months. (**A**) Sankey diagram showing basic adherence to guideline-directed medical therapy, from patients’ first visit to the HFC, to their most-recent visit. (**B**) The proportion of patients reaching target dose, at ≥ 50%-99% of target dose, or on another dose and/or a dose not captured, as of the most recent-visit to the HFC. ACEI, angiotensin-converting enzyme inhibitor; ARB, angiotensin-receptor blocker; ARNI, angiotensin receptor–neprilysin inhibitor; βb, beta-blocker; MRA, mineralocorticoid-receptor antagonist.∗For perindopril only, ≥ 50%-99% dose was not captured.

**Figure 2:**
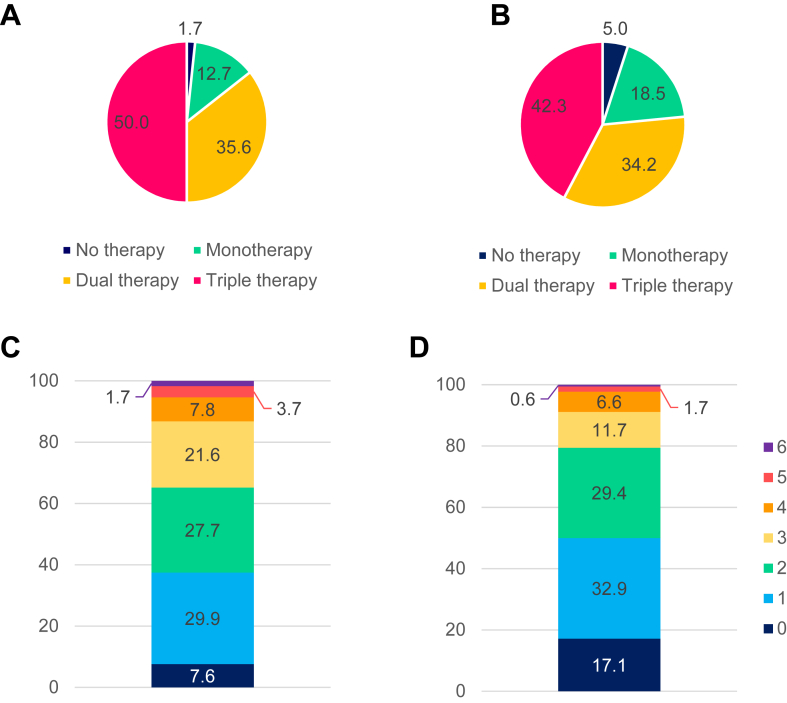
Comparison of patients with heart failure with reduced ejection fraction for those who were (**left**) followed at the heart failure clinic (HFC) for ≥6 months and (**right**) patients new to the HFC. The proportion of patients on no therapy, monotherapy, dual therapy, and triple therapy for (**A**) those who were followed for ≥6 months at the HFC and (**B**) those who were new patients. Panels (**C** and **D**) are the proportion of a subgroup of patients who had complete dosing information for the modified heart failure collaboratory score (see Supplemental Table S1 for definition): (**C**) those who were followed for ≥6 months at the HFC; and (**D**) those new to the HFC. The groups 0 to 6 represent those with a score of 0 points, 1 point and so on up to 6 points, which is the greatest number of points a patient can have for the scoring system. The Y axis for C and D represent the proportion of patients in each of the group in Figure 2, C and D.

A Sankey diagram showing the proportions of patients receiving each class of medical therapy, at their first visit to the HFC, and at their most-recent visit to the HFC, is shown in Figure 1A. The volume of prescription of ACEis and/or ARBs decreased from the initial visit to the most-recent visit, from 72.8% to 46.8%, whereas the volume of prescription of other classes of GDMT increased, as follows: ARNIs, from 4.1% to 34.4%; βbs, from 84.9% to 93.2%; and MRAs, from 43.6% to 58.8%. Slightly higher proportions of patients initiated on ACEis and MRAs discontinued GDMT, compared to the proportion on other medical therapies. Indication-corrected adherence also was calculated, based on available estimated glomerular filtration rate and potassium values, which slightly improved adherence rates (Table 3). Most patients were symptomatic, based on NYHA classifications (n = 613; 85.6%). Few (1.7%) were on no GDMT; 12.7% were on monotherapy; 35.6% were on dual therapy; and half (50.0%) were on triple therapy (Fig. 2A). A total of 242 patients (33.8%) had an LVEF value > 40% at their most-recent visit and could be considered to have HF with improved ejection fraction.

**Table 3:** Guideline-directed medical therapy for patients with heart failure with reduced ejection fraction

Patients	ACEi	ARB	ARNI	ACEi and/or ARB and/or ARNI	βb	MRA
**Followed for ≥ 6 mo at HFC, n = 716**						
**First visit to HFC**						
Basic adherence	420 (58.7)	101 (14.1)	29 (4.1)	550 (76.8)	608 (84.9)	312 (43.6)
**Most-recent visit to HFC**						
Basic adherence	237 (33.1)	98 (13.7)	246 (34.4)	581 (81.1)	667 (93.2)	421 (58.8)
Ineligible due to eGFR/K+	20	11	12	43	N/A	27
Indication-corrected adherence	257 (35.9)	109 (15.2)	258 (36.0)	624 (87.2)	N/A	448 (62.6)
**New to HFC, n = 584**						
Basic adherence	248 (42.5)	71 (12.2)	127 (21.7)	446 (76.4)	508 (87.0)	289 (49.5)
Ineligible due to eGFR/K+	17	4	3	24	N/A	35
Indication-corrected adherence	265 (45.4)	75 (12.8)	130 (22.3)	470 (80.5)	N/A	324 (55.5)

Values are n (%).

ACEi, angiotensin-converting enzyme inhibitor; ARB, angiotensin-receptor blocker; ARNI, angiotensin receptor–neprilysin inhibitor; βb, beta-blocker; eGFR, estimated glomerular filtration rate; HFC, heart failure clinic; N/A, not available; MRA, mineralocorticoid-receptor antagonist.

At their most-recent visit to the HFC, more than one-third of patients initiated on ARNIs or βbs were at the guideline-recommended target dose (36.6% and 34.6%, respectively), or were at ≥ 50%-99% of the target dose (ARNI, 30.1%; βb, 40.5%). Conversely, the majority of patients initiated on ACEis and/or ARBs (62.7%), and MRAs (64.4%) were either on another dose or had dosing information that was not captured.

Complete dosing information was available for 462 patients within this cohort, for whom a modified HF collaboratory score was calculated (Supplemental Table S1; Fig. 2C). The vast majority of patients (n = 366; 79.2%) had a score of 1-3, indicative of their receiving triple therapy but at a lower dose than the target dose, or at the target dose but not receiving all 3 classes of GDMT. A minority of patients (n = 61; 13.2%) achieved a score of 4-6, corresponding to > 1 class of GDMT, and achievement of target dosing in ≥ 1 class. A low proportion of patients had a score of zero, corresponding to their receiving either no GDMT or dosages < 50% of the target dose (n = 35; 7.6%).

#### Patients new to HFCs

Patients were classified as being new to the HFC (n = 584; 44.7%) if they had been followed for < 6 months (n = 325), or if their most-recent visit to the HFC was also their initial visit to the HFC (n = 259). As of their most-recent visit, the following proportions of patients had been given a prescription for the indicated medication: 54.6%, ACEis and/or ARBs; 21.7%, ARNIs; 87.0%, βbs; and 49.5%, MRAs. The proportions of patients on no GDMT (3.1%), monotherapy (17.5%), dual therapy (33.8%), or triple therapy (45.5%) were relatively similar to those of patients who had been followed for ≥ 6 months at the HFC, although a slightly higher proportion of patients were on either no therapy or monotherapy. However, a smaller proportion of patients (n = 106; 18.2%) had an LVEF value > 40% (HF with improved ejection fraction) at their most-recent visit (Fig. 2B).

Complete dosing information for patients new to the HFC was available for 350 patients, for whom an HF collaboratory score was calculated (Fig. 2D). A comparatively higher proportion of patients new to the HFC had an HF collaboratory score of zero (n = 60; 17.1%), relative to the proportion of those who had been followed at the HFC for ≥ 6 months, whereas a similar proportion of patients had a score in the 1-3 range (n = 259; 74.0%; either triple therapy at lower than target dose, or at target dose but not on all 3 classes of GDMT), or a score of 4-6 (n = 31; 8.9%; achieving target dose in > 1 class of GDMT).

### Prescription of HF medical therapy in patients with HFpEF

**Figure 3:**
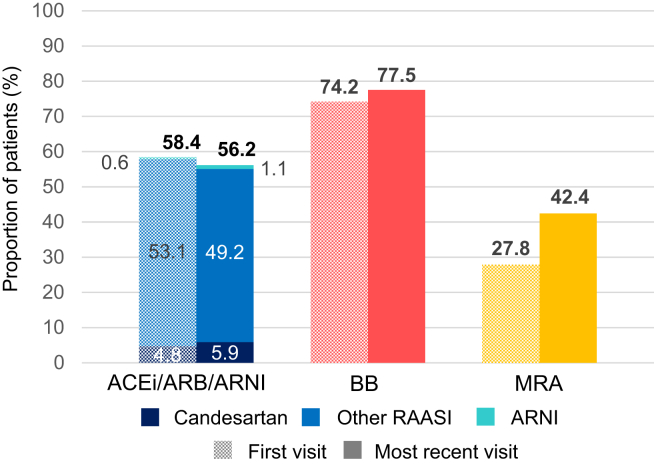
Medical-therapy prescription for patients with heart failure with preserved ejection fraction. The proportion of patients on each class of medication at their (**left, shaded bar**) first visit to the heart failure clinic, and (**right, solid bar**) their most-recent visit to the heart failure clinic. The group of those patients on angiotensin-converting enzyme inhibitor (ACEi) and/or angiotensin-receptor blocker (ARB) and/or angiotensin receptor–neprilysin inhibitor (ARNI) was broken down further into those on candesartan (as it is the only medical therapy within this class that was recommended by the 2017 Canadian Cardiovascular Society guidelines), vs those on other ACEis and/or ARBs, vs those on ARNIs. BB, beta-blocker; MRA, mineralocorticoid-receptor antagonist; RAASI, renin–angiotensin–aldosterone system inhibitor.

As of their most-recent HFC visit, ACEis and/or ARBs and/or ARNIs, and βbs and MRAs, were prescribed for 49.2% (including 1.1% who were prescribed ARNIs), 77.5%, and 42.4% of patients, respectively (Fig. 3). Candesartan was prescribed to only 5.9% (n = 21) patients. Overall, rates of prescription of medical therapy for existing patients at the HFC with HFpEF were similar from their first visit to their most-recent visit, except for MRAs, for which the proportion who had prescriptions increased from 27.8% at the first visit to 42.4% at the most-recent visit.

## Discussion

As all patients in the CAN-HF Registry had their most-recent HFC visit between 2017 and 2020, GDMT use must be contextualized within the 2017 CCS HF guidelines, which recommend that (absent contraindications) most patients with HFrEF be treated with “triple therapy,” including an ACEi (or an ARB for ACEi-intolerant patients), a βb, and an MRA.^13^ The guidelines further recommend that eligible ACEi and/or ARB patients who remain symptomatic following initiation of triple therapy be switched to an ARNI, and that ivabradine be added for symptomatic patients in sinus rhythm who have a heart rate of ≥ 70 beats per minute.

Our data demonstrate that for patients who were followed at the HFC for ≥ 6 months, prescription of each class of GDMT improved between their first visit and their most-recent visit. The most-notable changes were an increase of > 30% in the proportion of patients prescribed ARNIs, and an increase of > 15% in the proportion of patients prescribed an MRA. However, > 85% of these patients remained symptomatic, based on NYHA classification. Thus, the fact that almost half of patients remained on an ACEi and/or ARB, given the demonstrated superior effectiveness of sacubitril/valsartan, vs enalapril, in HFrEF outpatients, is surprising.^15^ In addition, projections suggest that a substantial number of deaths, hospitalizations, and HF readmissions in Canada potentially could be avoided by sacubitril/valsartan optimization.^16^ Factors related to the prescription rates for ARNIs were not assessed, but they may include clinical inertia, other clinical factors, such as renal dysfunction and/or hypotension and/or hyperkalemia, as well as access to the medication at the time of the study. Likewise surprising was the finding that roughly 40% of patients had not been initiated on an MRA, given that only 27 patients (3.8%) were ineligible based on their most-recent laboratory potassium values and that the medication was widely accessible at the time of data collection.

Our data further showed that fewer patients who had been managed at the HFC for > 6 months were on no GDMT, or were on monotherapy, compared to the number who were new to the clinic, and more patients were on dual or triple therapy. The modified HFC collaboratory scores further underscored this finding; they also indicate that those managed at the HFC for > 6 months were on higher doses of GDMT.

Determinants of GDMT-adherence gaps in specialized HF-care settings include limiting physiological factors, clinical inertia, nonmedical factors such as medication costs and access, and limited access to healthcare facilities or specialists.^17^, ^18^, ^19^ In a recent Ontario-based study,^20^ the main limitations preventing optimal management of patients with HF were lack of resources (personnel, communication with other physicians and/or specialists, and time), and lack of education and/or training and/or experience in HF management. Lack of experience and education and/or training were related mainly to unfamiliarity with HF guidelines, current drug therapy, and medication management for patients with HF and comorbidities. The perception that patients are “stable” also is hypothesized to contribute to clinical inertia and has led to calls in recent years to avoid use of this term, given the residual risk of sudden cardiac death or hospitalization, and the fact that optimization of GDMT can reduce this risk.^21^^,^^22^

Comparisons of our data to those from other retrospective analyses of patients with HFrEF suggest that the prescription rates of GDMT in specialized HFCs are higher. A recent Canadian study analyzed the use of GDMT over a 6-month period, following index hospitalization for HF, in a large cohort of patients aged ≥ 65years.^23^ That study found that approximately 20% of patients with HFrEF were receiving triple therapy, in contrast to almost half of outpatients with HFrEF in the CAN-HF Registry (irrespective of the length of follow-up at the HFC). Reasons for this discordance may be related to the time frame of data collection (2013-2018, vs 2017-2020 in the CAN-HF Registry); additionally, the CAN-HF study analyzed GDMT prescription for patients managed at HFCs by medical personnel trained in the management of HF, and the age of participants was not limited.

Likewise, prescription rates for GDMT were higher in the present study than they were in the Change the Management of Patients with Heart Failure (CHAMP-HF) study, a US-based registry study of 3518 HFrEF patients in mainly community cardiology and primary-care practices.^8^ Treatment rates of 69% for ACEis and/or ARBs and/or ARNIs, 67% for βbs, and 33% for MRAs were documented in the CHAMP-HF study; overall, these were lower for each class of medication than the rates documented for outpatients in the CAN-HF study. The proportion of patients achieving targets doses for ACEis and/or ARBs, ARNIs, and βbs was also considerably lower in the CHAMP-HF study, compared to that in our study, despite the use of a similar definition of target dose.

In the CHAMP-HF study, a practice-level variation in care occurred; cardiologists provided greater GDMT optimization. Other studies have demonstrated that patients attended by cardiologists have better outcomes than do those attended by noncardiologists,^24^, ^25^, ^26^ and that the level of GDMT use is higher, and target doses are achieved more rapidly, in HFCs as compared to other outpatient cardiology clinics.^27^ The data from the present study, along with the findings from these other studies, support the presence of need for more-specialized care. Other studies have demonstrated that collaborative care between primary-care physicians and cardiologists results in better care than that provided by physicians working in isolation.^28^^,^^29^ Currently, access is limited to more-specialized HF care, with only 15% of Canadian patients obtaining more-specialized care.^30^ Thus, a need is present to develop a system that would provide coordinated and better access to specialized care for this patient population.^31^

Comparisons of our data to those of other Canadian studies evaluating prescription of GDMT in specialized HFCs have yielded mixed results. Outpatients in the CAN-HF study had a lower level of prescription of GDMT, relative to that in a 2017 study of 511 patients at a single Canadian academic hospital–based multidisciplinary HFC, which documented high rates of GDMT use across all classes of medication (ie, ACEi and/or ARB, 83%; βbs, 99%; MRAs, 93%; ARNIs, 91%).^17^ This discrepancy was evident, even after evaluation of indication-corrected adherence. Conversely, prescription rates for MRAs (32%), and to a lesser extent, for renin–angiotensin–aldosterone system inhibitors (67%), were lower, in a recent study of 3 HFCs in the province of British Columbia^32^ that evaluated the use of GDMT in patients with HFrEF, aged ≥ 80 years, than those we observed in our study.

Our report is one of few contemporary accounts describing treatment of ambulatory patients with HFpEF. Adherence to GDMT is limited by the paucity of evidence at the time of this study, with only weak recommendations, in the CCS 2017 HF guidelines, for treatment with candesartan or an MRA.^13^ The evidence for βb use in this population is limited, with previous studies showing neutral effects.^33^^,^^34^ Use of βbs in this population in the CAN-HF study may be attributable to comorbidities, such as atrial fibrillation or CAD.

### Study limitations

Data from the CAN-HF study represent patients from sites that elected to participate in the registry, and they may not be generalizable to all care practices. Past intolerances, degree of frailty, physiological parameters limiting treatment initiation, and contraindications to GDMT were not captured, all of which may affect medication use. Specifically, heart rate and systolic blood pressure data were not collected for outpatients included in the CAN-HF Registry; as a result, these parameters may have limited the initiation of GDMT. Complete dosing information also was not available for all outpatients in the CAN-HF study, limiting the conclusions that may be drawn from our data set. The CAN-HF Registry also did not follow patients over time. As with many registry studies, CAN-HF Registry data were based on available documentation within patient medical records; thus, the data reported in the present analysis are constrained by this limitation.

### Conclusion

We observed an improvement in prescription of GDMT in patients managed at a HFC for > 6 months, between their first visit to the clinic and their most-recent visit. Although the most-notable increases occurred in prescription rates for ARNIs and MRAs, many patients who were symptomatic based on their NYHA class were not switched from ACEis and/or ARBs to ARNIs, and were not prescribed MRAs per guideline recommendations. Given that recent network meta-analyses have estimated that substantial reductions in both cardiovascular and all-cause mortality, as well as HF hospitalizations, can be achieved with incremental use of GDMT in combination with other HF medications,^35^^,^^36^ medication optimization in clinical practice should be prioritized in line with HF guideline recommendations. Our data from HFCs, affiliated in most cases with academic centres, compare favourably with those of other analyses of ambulatory patients with HFrEF, supporting a specialized patient-care model. Additionally, we describe real-world treatment of Canadian outpatients with HFpEF, documenting that few patients were prescribed candesartan and less than half of patients were on an MRA, per (weak) guideline recommendations.
