# Supplementary material for: The Canadian Heart Failure (CAN-HF) Registry: A Canadian Multicentre, Retrospective Study of Outpatients with Heart Failure
Source: CJC Open. 2024 Oct 9;7(1):1–9. doi: 10.1016/j.cjco.2024.09.014 (PMC11763239; doi:10.1016/j.cjco.2024.09.014)
Supplement: Supplemental Figure S1 and Table S1 [file mmc1.pdf]

Supplemental Figure S1. Patient flow diagram

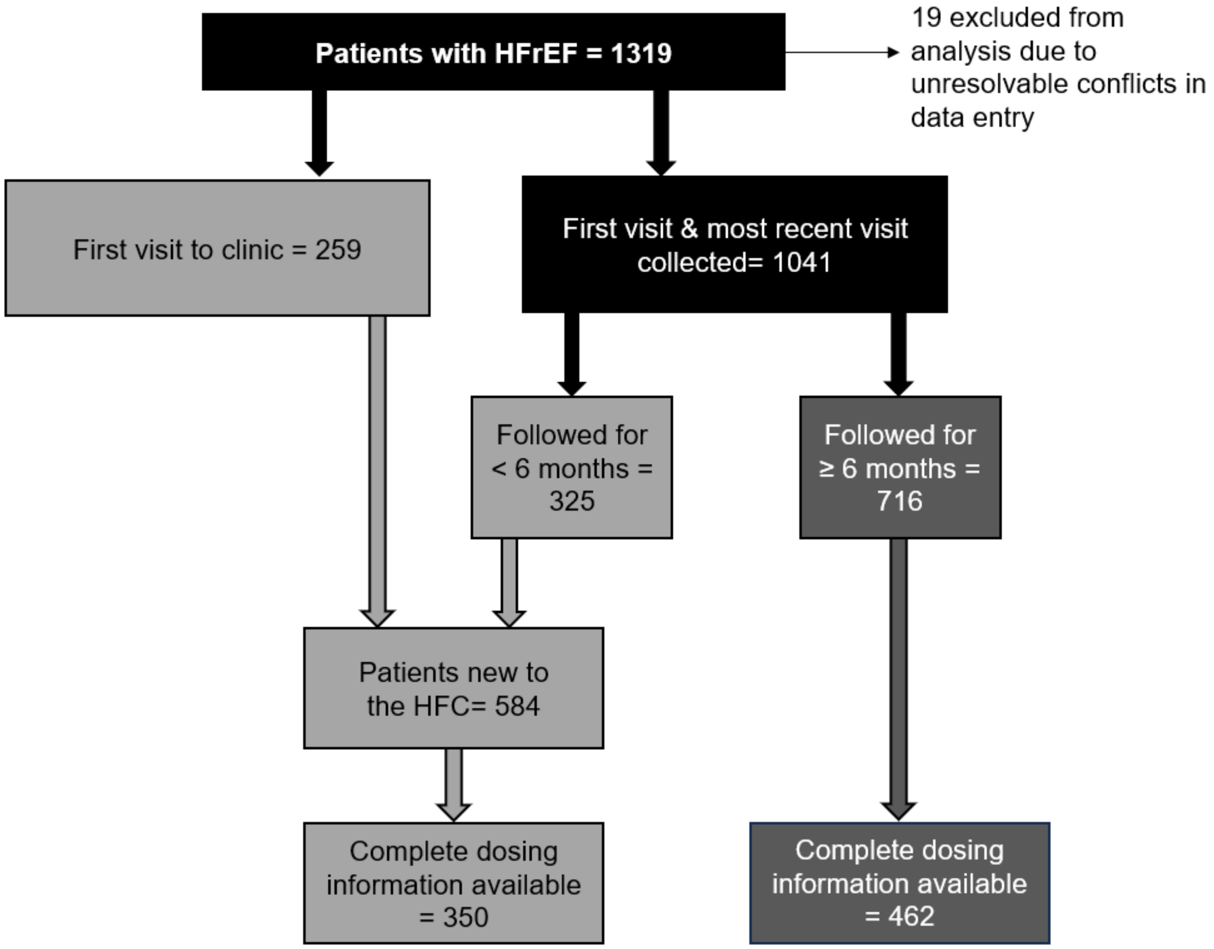

Supplemental Table S1. Modified HF Collaboratory Score

	No dose	≥50-99% of target dose	At target dose
ACEi/ARB/ARNI*	0	1	2
βb	0	1	2
MRA	0	1	2
Ivabradine	0	1**	N/A
Hydralazine/nitrates	0	1**	N/A

*For patients who were symptomatic based on NYHA (i.e. NYHA ≥2), 2 points were assigned if the patient was at target dose of ARNI, 1 point was assigned if the patient was on any other dose of ARNI, and 0 points were assigned if the patient was on ACEi/ARB.
**For Ivabradine and hydralazine/nitrates, any dosage was assigned 1 point.
